# The relationship between sleep duration and all-cause mortality in the older people: an updated and dose-response meta-analysis

**DOI:** 10.1186/s12889-020-09275-3

**Published:** 2020-07-28

**Authors:** Mengyang He, Xiangling Deng, Yuqing Zhu, Luyao Huan, Wenquan Niu

**Affiliations:** 1 Graduate School, Beijing University of Chinese Medicine, Beijing, China; 2 International Medical Department, China-Japan Friendship Hospital, Beijing, China; 3 Institute of Clinical Medical Sciences, China-Japan Friendship Hospital, No.2 Yinghua East Street, Chao Yang District, Beijing, 100029 China

**Keywords:** All-cause mortality, Meta-analysis, Older people, Sleep duration

## Abstract

**Background:**

Short or long sleep duration is proposed as a potential risk factor for all-cause mortality in the older people, yet the results of published studies are not often reproducible.

**Methods:**

Literature retrieval, study selection and data extraction were completed independently and in duplicate. Only prospective cohort studies were included. Effect-size estimates are expressed as hazard ratio (HR) and 95% confidence interval (CI).

**Results:**

Summary data from 28 articles, involving a total of 95,259 older people, were meta-analyzed. Overall analyses revealed a remarkably significant association between long sleep duration and all-cause mortality (adjusted HR = 1.24, 95% CI: 1.16–1.33, *P* < .001), whereas only marginal significance was observed for short sleep duration (adjusted HR = 1.04; 95% CI: 1.00–1.09; *P* = .033). Funnel plots suggested no publication bias for short sleep duration (*P* = .392). The probability of publication bias was high for long sleep duration (*P* = .020), yet the trim-and-fill method strengthened its significance in predicting all-cause mortality. In subgroup analyses, the association of long sleep duration with all-cause mortality was statistically significant in both women (HR = 1.48; 95% CI: 1.18–1.86; *P* = .001) and men (HR = 1.31; 95% CI: 1.10–1.58; *P* = .003). By contrast, with regard to short sleep duration, statistical significance was observed in men (HR = 1.13; 95% CI: 1.04–1.24; *P* = .007), but not in women (HR = 1.00; 95% CI: 0.85–1.18; *P* = .999) (Two-sample Z test *P* = .099). Besides gender, geographic region, sleep survey method, baseline age and follow-up interval were identified as possible causes of between-study heterogeneity in subgroup analyses. Further dose-response regression analyses revealed that trend estimation was more obvious for long sleep duration (regression coefficient: 0.13; *P* < .001) than for short sleep duration (regression coefficient: 0.02; *P* = .046).

**Conclusions:**

Our findings indicate a significantly increased risk of all-cause mortality associated with long sleep duration, especially in women, as well as with short sleep duration in men only.

## Background

It is widely recognized that sleep plays an important role in human mental and physical health [1, 2]. Experimental studies indicated that sleep deprivation and excessive sleep duration can exert an adverse effect on hormones, metabolism and immune function [3–5]. From epidemiological aspects, although dozens of studies reported that inappropriate sleep duration and poor sleep quality are reported to be associated with high risk of some common diseases, including diabetes [6], cardiovascular diseases [7] and cancer [8], as well as to increased all-cause and cause-specific mortality rates [9], these associations are not often reproducible.

Over the past decades, many prospective studies have reported a U-shaped relationship between sleep duration and all-cause mortality, with the nadir at 7–8 h of sleep per night [10–17]. In 2016, da Silva and colleagues conducted a meta-analysis by pooling the results of 27 cohort studies, and they found a significant association of both long and short sleep duration with increased all-cause mortality risk in the older people, and the association was more evident for long sleep duration [18]. However, the results of other studies have failed to provide any supportive data on sleep duration and mortality in the older people [19–21]. The reasons for these inconsistent reports are multifactorial, possibly relating to inadequate statistical power of individual studies, different backgrounds and characteristics of study groups, and lack of adjustment for confounding factors. Given the accumulating data afterwards, there is a need to reexamine this association in a more comprehensive manner.

To yield more information for future studies, we synthesized the results of prospective cohort studies in the older people, aiming to evaluate the association between sleep duration and all-cause mortality. Meanwhile, we also intended to explore possible causes of between-study heterogeneity.

## Methods

This meta-analysis was conducted according to the guidelines of the Preferred Reporting Items for Systematic Reviews and Meta-analyses (PRISMA) statement [22], and the PRISMA checklist is presented in Supplementary Table 1.

### Search strategy

We completed literature search by scanning PubMed, EMBASE and Web of Science databases as of November 30, 2019. The following medical topic terms are used: (sleep OR sleep disorders OR sleep duration OR drowse OR napping OR naps OR nap OR Siesta OR drowsiness OR drowse OR insomnia OR actigraphy sleep OR self-reported sleep [Title/Abstract], AND mortality OR death OR deaths OR premature death OR all-cause mortality [Title/Abstract]), AND (aged OR geriatrics OR older people OR older age OR older adult OR older adult OR older persons OR older people OR older men OR older women OR aging OR aging women OR aging men OR the older people OR aging individuals [Title/Abstract]). We also scanned the reference lists of retrieved articles and systematic reviews to avoid potential missing hits.

Two investigators (M.H. and X.D.) independently reviewed all retrieved articles, and, they carefully evaluated preliminary qualification based on their titles or abstracts and full texts if necessary.

### Inclusion/exclusion criteria

Our analyses were restricted to the articles that met the following criteria: (1) participants aged ≥60 years old; (2) all-cause mortality as the outcome; (3) prospective cohort studies; (4) clear classification of sleep duration; (5) at least 70% follow-up rate. Studies with subgroup analysis in older people on sleep duration and all-cause mortality were also included in this meta-analysis. Articles were excluded if they focused on cause-specific mortality or involved participants with serious diseases, or if they are case reports/series, editorials, and narrative comments.

### Data extraction

Two investigators (M.H. and X.D.) independently extracted data from each qualified article, and typed them into a standardized Excel spreadsheet, including name of the first author, year of publication, country where study was conducted, race, sample size, sex, baseline age, follow-up period, ascertainment of sleep duration, death certificate, adjusted confounders, sleep duration, effect estimation, and other traditional risk factors, if available. The divergences were resolved through joint reevaluation of original articles, and, if necessary, by a third author (W.N).

### Statistical analysis

We used the Stata software version 14.1 for Windows (Stata Corp, College Station, TX) to manage and analyze data. Irrespective of the magnitude of between-study heterogeneity, the random-effects model was employed. Effect size estimates are expressed as hazard ratio (HR) and its 95% confidence interval (CI), and the difference between two estimated was tested by the Z-test as reported by Altman and Bland [23]. The dose-response association was examined by the generalized least squares regression proposed by Greenland and Longnecker [24] for trend estimation of summarized dose-response data. Additionally, the restricted cubic splines of exposure distribution with 3 knots (25th, 50th, and 75th percentiles) were used to conduct nonlinearity test between sleep duration and all-cause mortality.

The inconsistency index (*I*^2^) is used to assess heterogeneity between studies, and it represents the percentage of diversity observed between studies that results from chance rather than an accidental result. If the *I*^2^ value is greater than 50%, significant heterogeneity is recorded, and a higher value indicates a higher degree of heterogeneity. Because of diverse sources of heterogeneity possibly from clinical and methodological aspects, a large number of prespecified subgroups were analyzed according to baseline age, sex, region, race, follow-up, short sleep duration and long sleep duration, respectively.

The probability of publication bias was evaluated by both Begg’s funnel plots and Egger regression asymmetry tests at a significance level of 10%. The trim-and-fill method was used to estimate the number of theoretically missing studies.

## Results

### Eligible studies

**Fig. 1:**
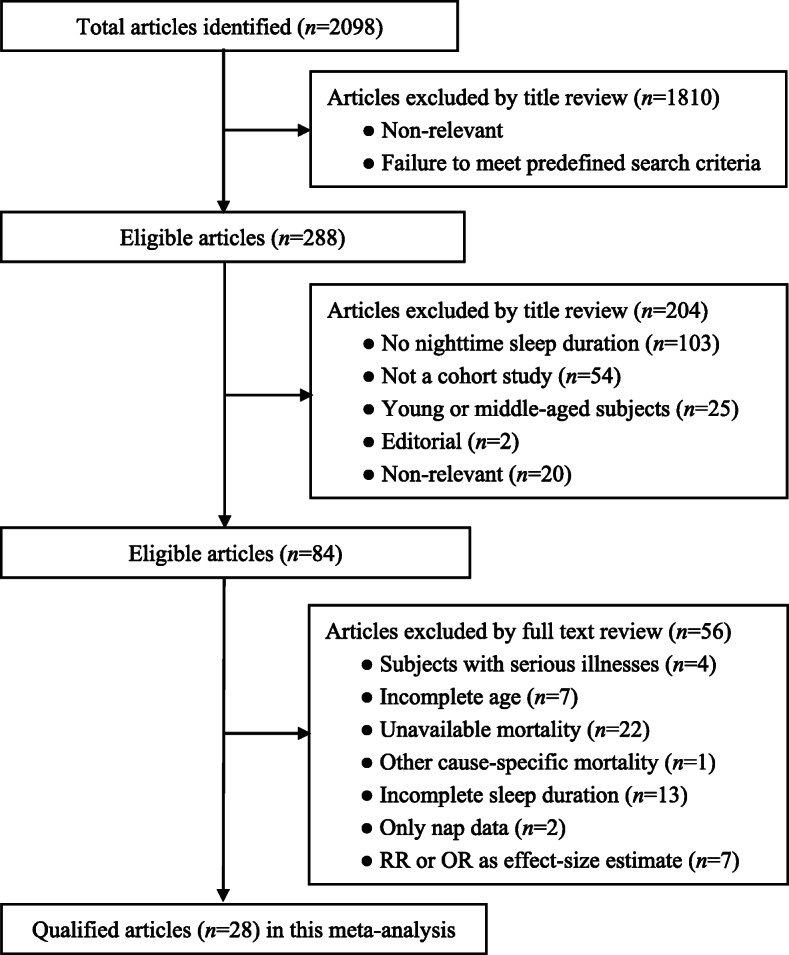
Flow chart of records retrieved, screened and included in this meta-analysis

After searching prespecified public databases using predefined medical subject terms, a total of 2098 articles were initially identified, and 28 of them with data on sleep duration and all-cause mortality were eligible for inclusion [10, 14, 17, 19, 21, 25–47], including 95,259 older persons in the final analysis. The detailed selection process including specific reasons for exclusion is schematized in Fig. 1. Since most articles provided data according to different age groups at baseline or follow-up periods, they are processed separately in subgroup analyses.

### Study characteristics

Table 1 and Table 2 show the baseline characteristics of all cohort studies involved in this meta-analysis. Of 28 eligible articles, 2 in older women [17, 19], and 6 specifically described the number of men and women and the number of deaths of men and women [27, 30, 35, 38–40]. Five articles provided data on the association between sleep duration and all-cause mortality by gender [30, 35, 36, 38, 42]. Of all eligible articles, 9 investigated the total sleep duration of 24 h in the older people [19, 26, 31, 33, 38, 39, 41–43], and the others focused on the nighttime. One article adopted the actigraphy method to collect sleep time [43], and 2 articles simultaneously used actigraphy method and questionnaires [17, 47]. Based on geographic locations, all eligible articles were classified into America [14, 17, 19, 26, 32, 33, 40, 42], Europe [10, 21, 34, 37, 42–45], and Asia [27–31, 35, 36, 38, 39, 41].

**Table 1:** The baseline characteristics of all cohort studies involved in this meta-analysis

First author (year)	Baseline year	Country	Age (years)	Ascertainment of sleep	TST	Comparison	Mortality ascertainment	Adjustment
Kaplan (1987)	1965	USA	60–94	Questionnaire	Nighttime sleep	7-8 h vs. ≤7 h	Death certificate	YES
						7-8 h vs. > 8 h	All-cause	
Seki (2001)	1990	Japan	60–74	Questionnaire	24 h sleep	7 h vs. ≤6 h	Death certificate	YES
						7 h vs. ≥9 h	All-cause	
Goto (2003)	1987	Japan	≥65	Questionnaire	Nighttime sleep	6-7 h vs. < 6 h	Death certificate	YES
						6-7 h vs. ≥7 h	All-cause	
Lan (2007)	1993	China	≥64	Questionnaire	Nighttime sleep	7–7.9 h vs. < 7 h	Death certificate	YES
						7–7.9 h vs. ≥10 h	All-cause, CVD, cancer	
Gangwisch (2008)	1982	USA	60–86	Questionnaire	Nighttime sleep	7 h vs. ≤5 h	Death certificate and proxy interviews	YES
						7 h vs. ≥9 h	All-cause	
Stone (2009)	1986	USA	≥68	Questionnaire	Nighttime sleep	6-8 h vs. < 6 h	Death certificate	YES
						6-8 h vs. ≥8 h	All-cause, CVD, cancer and other	
Suzuki (2009)	1999	Japan	65–85	Questionnaire	24 h sleep	7 h vs. ≤5 h	Death certificate	YES
						7 h vs. ≥10 h	All-cause and CVD	
Castro-Costa (2011)	1997	Brazil	> 60	Questionnaire	24 h sleep	7–7.9 h vs. < 6 h	Death certificate and proxy interviews	YES
						7–7.9 h vs. ≥9 h	All-cause	
Kripke (2011)	1995	USA	60–81	Questionnaire & actigraphy	Nighttime sleep	7 h vs. ≤5 h	Proxy interviews and social security death index	NO
						7 h vs. ≥9 h	All-cause	
Kronholm (2011)	1972	Finland	60–64	Questionnaire	Nighttime sleep	7-8 h vs. ≤5 h	Death certificate and hospital discharge register	NO
						7-8 h vs. ≥10 h	All-cause and CVD	
Qiu (2011)	2005	China	≥65	Questionnaire	24 h sleep	6-8 h vs. ≤5 h	Death certificate	YES
						6-8 h vs. ≥9 h	All-cause	
Werle (2011)	1994	Brazil	≥80	Questionnaire	Nighttime sleep & 24 h sleep	≤8 h vs. > 8 h	Death certificate, proxy interviews and patient records	YES
							All-cause and CVD	
Cohen-Mansfield (2012)	1989	Israel	75–94	Questionnaire	Nighttime sleep	7-9 h vs. < 7 h	Death certificate	YES
						7-9 h vs. ≥9 h	All-cause	
Chen (2013)	1999	China	> 65	Questionnaire	Nighttime sleep	7 h vs. ≤4 h	Death certificate	YES
						7 h vs. ≥9 h	All-cause, CVD, cancer	
Jung (2013)	1984	USA	60–96	Questionnaire	Nighttime sleep	7-9 h vs. < 6 h	Death certificate or notice from a family member or published obituary	YES
						7-9 h vs. ≥9 h	All-cause	
Kakizaki (2013)	1994	Japan	≥70	Questionnaire	24 h sleep	7 h vs. ≤6 h	Death certificate	YES
						7 h vs. ≥10 h	All-cause, CVD, cancer and the other	
Kim (2013)	1990	USA	≥65	Questionnaire	24 h sleep	7 h vs. ≤5 h	Death certificate	YES
						7 h vs. ≥9 h	All-cause and CVD	
Yeo (2013)	1993	Korea	≥60	Questionnaire	24 h sleep	7 h vs. ≤5 h	Death certificate	YES
						7 h vs. ≥10 h	All-cause, CVD, cancer	
Lee (2014)	2001	China	> 65	Questionnaire	Nighttime sleep	< 10 h vs. ≥10 h	Death certificate	YES
							All-cause	
Hall (2015)	–	USA	70–79	Questionnaire	Nighttime sleep	7 h vs. < 6 h	Death certificates, hospital records, informant interviews and autopsy data	YES
						7 h vs. > 8 h	All-cause	
Zuurbier (2015)	2004	Holland	60–98	Questionnaire & actigraphy	24 h sleep	6–7.5 hvs. < 6 h	Death certificate and patient records	YES
						6–7.5 hvs. > 7.5 h	All-cause	
Smagula (2016)	2003	USA	≥65	Questionnaire & actigraphy	24 h sleep	5-8 h vs. < 5 h	Death certificate	YES
						5-8 h vs. > 8 h	All-cause, CVD, cancer	
Akersted (2017)	1997	Swedish	≥65	Questionnaire	Nighttime sleep	7 h vs. ≤5 h	Death certificate	YES
						7 h vs. ≥8 h	All-cause, CVD, cancer	
Beydoun (2017)	2005	USA	≥65	Questionnaire	Nighttime sleep	7 h vs. < 7 h	Death certificate	YES
						7 h vs. > 8 h	All-cause	
Brostrom (2018)	2003	Sweden	65–82	Questionnaire	Nighttime sleep	7-8 h vs. ≤6 h	Death certificate	NO
						7-8 h vs. ≥9 h	All-cause	
Cheng (2018)	2009	Singapore	≥60	Questionnaire	Nighttime sleep	7-8 h vs. ≤6 h	Death certificate	YES
						7-8 h vs. ≥9 h	All-cause	
Akersted (2019)	1997	Swedish	≥65	Questionnaire	Nighttime sleep	7 h vs. ≤4 h	Death certificate,	YES
						7 h vs. ≥8 h	All-cause, CVD, cancer	
Morgan (2019)	1985	UK	≥65	Questionnaire	Nighttime sleep	7 h vs. ≤4 h	Death certificate	YES
						7 h vs. ≥9 h	All-cause	
						4–9.9 h vs. ≥10 h	All-cause	

Abbreviations: CVD, cardiovascular disease; TST, total sleep time

**Table 2:** The baseline characteristics of all cohort studies involved in this meta-analysis

Published year	First author	Gender	Sample size	Age	Men	Women	Follow up (years)	Total deaths	Men deaths	Women deaths	Exposure (h)	Ref (h)	Adjusted	HR, 95% CI
1987	Kaplan	Men, Women	4174	60–94	–	–	17	–	–	–	> 8	7–8	YES	1.02, 0.87–1.19
1987	Kaplan	Men, Women	4174	60–94	–	–	17	–	–	–	< 7	7–8	YES	1.02, 0.87–1.19
1987	Kaplan	Men, Women	4174	60–94	–	–	17	–	–	–	> 8	7–8	NO	1.02, 0.87–1.21
1987	Kaplan	Men, Women	4174	60–94	–	–	17	–	–	–	< 7	7–8	NO	1.02, 0.87–1.21
2011	Kronholm	Men, Women	1210	60–64	–	–	35	1065	–	–	≥10	7–8	NO	1.11, 1.05–1.18
2011	Kronholm	Men, Women	1210	60–64	–	–	35	1065	–	–	≤5	7–8	NO	1.07, 1.01–1.14
2008	Gangwisch	Men, Women	3983	60–86	–	–	10	1604	–	–	≥9	7	YES	1.36, 1.15–1.6
2008	Gangwisch	Men, Women	3983	60–86	–	–	10	1604	–	–	≤5	7	YES	1.27, 1.06–1.53
2008	Gangwisch	Men, Women	3983	60–86	–	–	10	1604	–	–	≥9	7	NO	1.98, 1.68–2.32
2008	Gangwisch	Men, Women	3983	60–86	–	–	10	1604	–	–	≤5	7	NO	1.72, 1.44–2.06
2013	Jung	Men	2001	60–96	889	1112	19	1224	632	592	≥9	7	YES	1.09, 0.82–1.45
2013	Jung	Women	2001	60–96	889	1112	19	1224	632	592	≥9	7	YES	1.51, 1.05–2.18
2013	Jung	Men	2001	60–96	889	1112	19	1224	632	592	≤5	7	YES	0.98, 0.67–1.43
2013	Jung	Women	2001	60–96	889	1112	19	1224	632	592	≤5	7	YES	1.11, 0.77–1.6
2013	Jung	Men	2001	60–96	889	1112	19	1224	632	592	≥9	7	NO	1.18, 0.92–1.52
2013	Jung	Women	2001	60–96	889	1112	19	1224	632	592	≥9	7	NO	1.50, 1.12–2.00
2013	Jung	Men	2001	60–96	889	1112	19	1224	632	592	≤5	7	NO	1.10, 0.79–1.55
2013	Jung	Women	2001	60–96	889	1112	19	1224	632	592	≤5	7	NO	1.07, 0.79–1.44
2019	Morgan	Men, Women	960	≥65	375	585	27	927	–	–	≥9	7	YES	1.18, 0.85–1.63
2019	Morgan	Men, Women	960	≥65	375	585	27	927	–	–	≤4	7	YES	1.08, 0.83–1.40
2019	Morgan	Men, Women	960	≥65	375	585	27	927	–	–	≥9	7	NO	1.40, 1.08–1.83
2019	Morgan	Men, Women	960	≥65	375	585	27	927	–	–	≤4	7	NO	1.02, 0.80–1.29
2009	Stone	Women	8101	≥68	0	8101	6.9	1922	0	1922	> 8	6–8	YES	1.16, 0.97–1.39
2009	Stone	Women	8101	≥68	0	8101	6.9	1922	0	1922	< 6	6–8	YES	1.02, 0.87–1.19
2009	Stone	Women	8101	≥68	0	8101	6.9	1922	0	1922	≥10	8–9	YES	1.58, 1.27–1.95
2009	Stone	Women	8101	≥68	0	8101	6.9	1922	0	1922	< 6	8–9	YES	0.95, 0.76–1.18
2003	Goto	Men	724	≥65	251	473	12	305	139	166	> 7	6–7	YES	1.54, 0.92–2.58
2003	Goto	Women	724	≥65	251	473	12	305	139	166	> 7	6–7	YES	1.40, 0.91–2.15
2003	Goto	Men	724	≥65	251	473	12	305	139	166	< 6	6–7	YES	1.29, 0.50–3.34
2003	Goto	Women	724	≥65	251	473	12	305	139	166	< 6	6–7	YES	2.62, 1.36–5.07
2003	Goto	Men	724	≥65	251	473	12	305	139	166	> 7	6–7	NO	1.62, 0.99–2.66
2003	Goto	Women	724	≥65	251	473	12	305	139	166	> 7	6–7	NO	1.60, 1.06–2.42
2003	Goto	Men	724	≥65	251	473	12	305	139	166	< 6	6–7	NO	1.42, 0.61–3.27
2003	Goto	Women	724	≥65	251	473	12	305	139	166	< 6	6–7	NO	2.65, 1.42–4.95
2012	Cohen-Mansfield	Men, Women	1166	≥75	–	–	20	1108	–	–	> 9	7–9	YES	1.32, 1.09–1.58
2012	Cohen-Mansfield	Men, Women	1166	≥75	–	–	20	1108	–	–	< 7	7–9	YES	0.98, 0.84–1.13
2012	Cohen-Mansfield	Men, Women	1166	≥75	–	–	20	1108	–	–	> 9	7–9	NO	1.29, 1.11–1.52
2012	Cohen-Mansfield	Men, Women	1166	≥75	–	–	20	1108	–	–	< 7	7–9	NO	0.81, 0.71–0.93
2013	Kim	Men, Women	–	65–69	–	–	12.9	4764	–	–	≥9	7	YES	1.25, 1.14–1.38
2013	Kim	Men, Women	–	65–69	–	–	12.9	4764	–	–	≤5	7	YES	1.13, 1.02–1.26
2013	Kim	Men, Women	–	≥70	–	–	12.9	6444	–	–	≥9	7	YES	1.14, 1.05–1.24
2013	Kim	Men, Women	–	≥70	–	–	12.9	6444	–	–	≤5	7	YES	1.09, 0.99–1.19
2001	Seki	Men, Women	1065	60–74	440	625	7.5	123	77	46	≥9	7	YES	0.97, 0.50–1.90
2001	Seki	Men, Women	1065	60–74	440	625	7.5	123	77	46	< 6	7	YES	1.74, 0.72–4.24
2001	Seki	Men, Women	1065	60–74	440	625	7.5	123	77	46	≥9	7	NO	1.00, 0.52–1.96
2001	Seki	Men, Women	1065	60–74	440	625	7.5	123	77	46	< 6	7	NO	2.17, 0.91–5.21
2007	Lan	Men	3079	≥64	1748	1331	10	1338	816	522	≥10	7–7.9	YES	1.51, 1.19–1.92
2007	Lan	Women	3079	≥64	1748	1331	10	1338	816	522	≥10	7–7.9	YES	2.06, 1.50–2.83
2007	Lan	Men	3079	≥64	1748	1331	10	1338	816	522	< 7	7–7.9	YES	0.98, 0.76–1.25
2007	Lan	Women	3079	≥64	1748	1331	10	1338	816	522	< 7	7–7.9	YES	1.14, 0.77–1.67
2007	Lan	Men	3079	≥64	1748	1331	10	1338	816	522	≥10	7–7.9	NO	1.86, 1.48–2.34
2007	Lan	Women	3079	≥64	1748	1331	10	1338	816	522	≥10	7–7.9	NO	2.49, 1.84–3.37
2007	Lan	Men	3079	≥64	1748	1331	10	1338	816	522	< 7	7–7.9	NO	0.97, 0.76–1.23
2007	Lan	Women	3079	≥64	1748	1331	10	1338	816	522	< 7	7–7.9	NO	1.04, 0.71–1.51
2013	Yeo	Men, Women	5538	≥60	–	–	9.4	1223	–	–	≥10	7	YES	1.48, 1.13–1.93
2013	Yeo	Men, Women	5538	≥60	–	–	9.4	1223	–	–	≤5	7	YES	1.23, 1.03–1.47
2013	Kakizaki	Men, Women	9690	≥70	–	–	10.8	3960	–	–	≥9	7–7.9	YES	1.33, 1.24–1.43
2013	Kakizaki	Men, Women	9690	≥70	–	–	10.8	3960	–	–	< 6	7–7.9	YES	0.98, 0.87–1.10
2011	Werle	Men, Women	187	≥80	68	119	8.7	141	56	85	> 8	7	YES	0.95, 0.89–1.02
2011	Werle	Men, Women	187	≥80	68	119	8.7	141	56	85	> 8	7	NO	0.95, 0.90–1.01
2011	Kripke	Women	355	60–81	–	–	10.5	79	–	–	≥9	7–7.9	NO	0.93, 0.37–2.35
2011	Kripke	Women	355	60–81	–	–	10.5	79	–	–	≤5	7	NO	0.83, 0.4–1.73
2017	Akersted	Men, Women	8089	≥65	3879	4210	13	2337	–	–	≥8	7	YES	1.01, 0.90–1.14
2017	Akersted	Men, Women	8089	≥65	3879	4210	13	2337	–	–	≤5	7	YES	1.05, 0.90–1.22
2017	Akersted	Men, Women	8089	≥65	3879	4210	13	2337	–	–	≥8	7	NO	1.06, 0.96–1.19
2017	Akersted	Men, Women	8089	≥65	3879	4210	13	2337	–	–	≤5	7	NO	1.02, 0.90–1.16
2011	Castro-Costa	Men, Women	1512	> 60	–	–	7.5	440	–	–	≥9	7–7.9	YES	1.56, 1.12–2.18
2011	Castro-Costa	Men, Women	1512	> 60	–	–	7.5	440	–	–	< 6	7–7.9	YES	0.88, 0.61–1.28
2011	Castro-Costa	Men, Women	1512	> 60	–	–	7.5	440	–	–	≥9	7–7.9	NO	1.84, 1.40–2.43
2011	Castro-Costa	Men, Women	1512	> 60	–	–	7.5	440	–	–	< 6	7–7.9	NO	1.01, 0.75–1.37
2013	Chen	Men, Women	4064	> 65	2269	1795	9	1004	336	668	≥9	7	YES	1.66, 1.28–2.17
2013	Chen	Men, Women	4064	> 65	2269	1795	9	1004	336	668	≤4	7	YES	1.00, 0.75–1.33
2009	Suzuki	Men, Women	11,395	65–85	5825	5570	7	1004	689	315	≥10	7	YES	1.96, 1.49–2.57
2009	Suzuki	Men, Women	11,395	65–85	5825	5570	7	1004	689	315	≤5	7	YES	0.92, 0.66–1.28
2009	Suzuki	Men	11,395	65–85	5825	5570	7	1004	689	315	≥10	7	YES	1.86, 1.34–2.56
2009	Suzuki	Women	11,395	65–85	5825	5570	7	1004	689	315	≥10	7	YES	2.27, 1.37–3.76
2009	Suzuki	Men	11,395	65–85	5825	5570	7	1004	689	315	≤5	7	YES	1.08, 0.72–1.61
2009	Suzuki	Women	11,395	65–85	5825	5570	7	1004	689	315	≤5	7	YES	0.71, 0.39–1.29
2009	Suzuki	Men, Women	11,395	65–85	5825	5570	7	1004	689	315	≥10	7	NO	2.29, 1.75–3.00
2009	Suzuki	Men, Women	11,395	65–85	5825	5570	7	1004	689	315	≤5	7	NO	1.03, 0.74–1.43
2009	Suzuki	Men	11,395	65–85	5825	5570	7	1004	689	315	≥10	7	NO	2.16, 1.57–2.98
2009	Suzuki	Women	11,395	65–85	5825	5570	7	1004	689	315	≥10	7	NO	2.65, 1.61–4.37
2009	Suzuki	Men	11,395	65–85	5825	5570	7	1004	689	315	≤5	7	NO	1.16, 0.78–1.73
2009	Suzuki	Women	11,395	65–85	5825	5570	7	1004	689	315	≤5	7	NO	0.82, 0.46–1.48
2014	Lee	Men	3427	> 65	1745	1682	5	297	221	76	≥10	< 10	YES	1.75, 1.09–2.81
2014	Lee	Women	3427	> 65	1745	1682	5	297	221	76	≥10	< 10	YES	2.88, 1.01–8.20
2014	Lee	Men	3427	> 65	1745	1682	5	297	221	76	≥10	< 10	NO	2.10, 1.33–3.33
2014	Lee	Women	3427	> 65	1745	1682	5	297	221	76	≥10	< 10	NO	2.70, 0.98–7.46
2018	Brostrom	Men	630	65–82	301	329	6	144	86	58	≥9	7–8	YES	1.10, 0.1–10.30
2018	Brostrom	Women	630	65–82	301	329	6	144	86	58	≥9	7–8	YES	0.35, 0.10–26.90
2018	Brostrom	Men	630	65–82	301	329	6	144	86	58	≤6	7–8	YES	0.60, 0.10–2.90
2018	Brostrom	Women	630	65–82	301	329	6	144	86	58	≤6	7–8	YES	0.34, 0.10–1.90
2016	Smagula	Men	2531	≥65	2531	0	7.4	628	628	0	> 8	5–8	YES	0.83, 0.71–1.31
2016	Smagula	Men	2531	≥65	2531	0	7.4	628	628	0	< 5	5–8	YES	1.12, 0.89–1.42
2016	Smagula	Men	2531	≥65	2531	0	7.4	628	628	0	> 8	5–8	NO	1.02, 0.76–1.37
2016	Smagula	Men	2531	≥65	2531	0	7.4	628	628	0	< 5	5–8	NO	1.28, 1.02–1.62
2015	Zuurbier	Men, Women	1073	60–98	–	–	7.3	142	–	–	> 7.5	6–7.5	YES	1.24, 0.73–2.10
2015	Zuurbier	Men, Women	1073	60–98	–	–	7.3	142	–	–	< 6	6–7.5	YES	1.12, 0.75–1.68
2011	Qiu	Men, Women	12,671	≥65	5421	7250	3	5199	2067	3132	≥10	8	YES	1.09, 1.00–1.180
2011	Qiu	Men, Women	12,671	≥65	5421	7250	3	5199	2067	3132	≤5	8	YES	0.97, 0.88–1.08
2011	Qiu	Men	12,671	≥65	5421	7250	3	5199	2067	3132	≥10	8	YES	1.22, 1.08–1.38
2011	Qiu	Women	12,671	≥65	5421	7250	3	5199	2067	3132	≥10	8	YES	1.00, 0.90–1.11
2011	Qiu	Men	12,671	≥65	5421	7250	3	5199	2067	3132	≤5	8	YES	1.17, 1.01–1.38
2011	Qiu	Women	12,671	≥65	5421	7250	3	5199	2067	3132	≤5	8	YES	0.85, 0.75–0.98
2011	Qiu	Men, Women	12,671	65–79	5421	7250	3	5199	2067	3132	≥10	8	YES	1.17, 0.88–1.54
2011	Qiu	Men, Women	12,671	65–79	5421	7250	3	5199	2067	3132	≤5	8	YES	1.00, 0.74–1.35
2011	Qiu	Men, Women	12,671	≥80	5421	7250	3	5199	2067	3132	≥10	8	YES	1.08, 0.99–1.18
2011	Qiu	Men, Women	12,671	≥80	5421	7250	3	5199	2067	3132	≤5	8	YES	0.97, 0.87–1.08
2011	Qiu	Men, Women	12,671	≥65	5421	7250	3	5199	2067	3132	≥10	8	NO	1.22, 1.13–1.32
2011	Qiu	Men, Women	12,671	≥65	5421	7250	3	5199	2067	3132	≤5	8	NO	1.19, 1.08–1.32
2011	Qiu	Men	12,671	≥65	5421	7250	3	5199	2067	3132	≥10	8	NO	1.36, 1.20–1.54
2011	Qiu	Women	12,671	≥65	5421	7250	3	5199	2067	3132	≥10	8	NO	1.12, 1.02–1.25
2011	Qiu	Men	12,671	≥65	5421	7250	3	5199	2067	3132	≤5	8	NO	1.47, 1.26–1.71
2011	Qiu	Women	12,671	≥65	5421	7250	3	5199	2067	3132	≤5	8	NO	1.03, 0.90–1.17
2011	Qiu	Men, Women	12,671	65–79	5421	7250	3	5199	2067	3132	≥10	8	NO	1.46, 1.11–1.91
2011	Qiu	Men, Women	12,671	65–79	5421	7250	3	5199	2067	3132	≤5	8	NO	1.32, 0.98–1.77
2011	Qiu	Men, Women	12,671	≥80	5421	7250	3	5199	2067	3132	≥10	8	NO	1.21, 1.12–1.32
2011	Qiu	Men, Women	12,671	≥80	5421	7250	3	5199	2067	3132	≤5	8	NO	1.18, 1.06–1.31
2017	Beydoun	Men, Women	2173	≥65	–	–	4.5	–	–	–	> 8	7–8	YES	1.30, 0.73–2.29
2017	Beydoun	Men, Women	2173	≥65	–	–	4.5	–	–	–	< 7	7–8	YES	0.96, 0.68–1.35
2017	Beydoun	Men, Women	2173	≥65	–	–	4.5	–	–	–	> 8	7–8	NO	1.90, 1.44–2.50
2017	Beydoun	Men, Women	2173	≥65	–	–	4.5	–	–	–	< 7	7–8	NO	1.20, 0.94–1.52
2018	Cheng	Men, Women	2448	≥60	1167	1281	4	274	–	–	≥9	7–8	YES	2.24, 1.05–4.77
2018	Cheng	Men, Women	2448	≥60	1167	1281	4	274	–	–	≤6	7–8	YES	2.14, 1.12–4.11
2018	Cheng	Men, Women	2448	≥60	1167	1281	4	274	–	–	≥9	7–8	NO	2.87, 1.36–6.05
2018	Cheng	Men, Women	2448	≥60	1167	1281	4	274	–	–	≤6	7–8	NO	2.69, 1.44–5.03
2015	Hall	Men, Women	3013	≥70	1463	1550	8.2	953	–	–	> 8	7	YES	1.23, 0.93–1.63
2015	Hall	Men, Women	3013	≥70	1463	1550	8.2	953	–	–	< 6	7	YES	1.06, 0.83–1.34
2015	Hall	Men, Women	3013	≥70	1463	1550	8.2	953	–	–	> 8	7	NO	1.49, 1.15–1.93
2015	Hall	Men, Women	3013	≥70	1463	1550	8.2	953	–	–	< 6	7	NO	1.30, 1.05–1.61
2019	Akersted	Men, Women	–	≥65	–	–	13	–	–	–	≥9	7	YES	0.99, 0.84–1.09
2019	Akersted	Men, Women	–	≥65	–	–	13	–	–	–	≤4	7	YES	0.97, 0.81–1.18
2019	Akersted	Men, Women	–	≥65	–	–	13	–	–	–	≥9	7	YES	0.91, 0.66–1.25

Abbreviations: Ref, reference; HR, hazard ratio; 95% CI, 95% confidence interval

### Quality assessment

Table 3 shows the quality assessment results by using the Newcastle-Ottawa Scale (NOS) tool for cohort studies, with the total scores (mean: 7.46, standard deviation: 0.74) ranging from 6 to 9 in this meta-analysis.

**Table 3:** The Newcastle-Ottawa Scale (NOS) for assessing the quality of all cohort studies involved in this meta-analysis

First author	Published year	Representative of the exposed cohort	Selection of the non-exposed cohort	Ascertainment of exposed	Demonstration that outcome of interest was no present at start of study	Control for important cohort	Additional factors	Assessment of outcome	Follow up	Adequacy of follow up	Score
Kaplan	1987	1	1	0	1	1	1	1	1	1	8
Seki	2001	1	1	0	1	1	1	1	1	1	8
Goto	2003	1	1	0	1	1	1	1	1	0	7
Lan	2007	1	1	0	1	1	1	1	1	0	7
Gangwisch	2008	1	1	0	1	1	1	1	1	0	7
Stone	2009	1	1	0	1	1	1	1	1	1	8
Suzuki	2009	1	1	0	1	1	1	1	1	1	8
Kronholm	2011	1	1	0	1	1	0	1	1	0	6
Werle	2011	1	1	0	1	0	1	1	1	0	6
Kripke	2011	1	1	1	1	1	0	1	1	1	8
Castro-Costa	2011	1	1	0	1	1	1	1	1	0	7
Qiu	2011	1	1	0	1	1	1	1	1	1	8
Cohen-Mansfield	2012	1	1	0	1	1	1	1	1	1	8
Jung	2013	1	1	0	1	1	1	1	1	1	8
Kim	2013	1	1	0	1	1	1	1	1	0	7
Yeo	2013	1	1	0	1	1	1	1	1	0	7
Kakizaki	2013	1	1	0	1	1	1	1	1	1	8
Chen	2013	1	1	0	1	1	1	1	1	1	8
Lee	2014	1	1	0	1	1	1	1	1	0	7
Zuurbier	2015	1	1	1	1	1	1	1	1	1	9
Hall	2015	1	1	0	1	1	1	1	1	0	7
Smagula	2016	1	1	1	1	1	1	1	1	1	9
Akersted	2017	1	1	0	1	1	1	1	1	0	7
Beydoun	2017	1	1	0	1	1	1	1	1	0	7
Brostrom	2018	1	1	0	1	1	0	1	1	1	7
Cheng	2018	1	1	0	1	1	1	1	1	0	7
Morgan	2019	1	1	0	1	1	1	1	1	1	8
Akersted	2019	1	1	0	1	1	1	1	1	0	7

### Overall analyses

After pooling the results of all qualified prospective cohorts together (Table 4), unadjusted effect-size estimates for the association of the long (HR = 1.43; 95% CI: 1.30–1.58; *P* < .001; *I*^2^ = 88.6%) and short (HR = 1.15; 95% CI: 1.06–1.25; *P* < .001; *I*^2^ = 71.5%) sleep duration with all-cause mortality in the older people were remarkably significant. After adjusting for potential confounders, long sleep duration was significantly associated with an increased risk of all-cause mortality in the older people (HR = 1.24; 95% CI: 1.16–1.33; *P* < .001), whereas only marginal significance was observed for short sleep duration (HR = 1.04; 95% CI: 1.00–1.09; *P* = .033) (Table 4). In view of the striking differences before and after adjustment, the following analyses are based on adjusted effect-size estimates for the sake of relative accuracy.

**Table 4:** Overall and subgroup analyses of short and long sleep duration with all-cause mortality in the older people

Group	Number of qualified studies	Short sleep duration		Long sleep duration	
		HR (95% CI); ***P***	***I***^**2**^	HR (95% CI); ***P***	***I***^**2**^
**Overall analyses**					
Mortality (unadjusted)	23/26	1.15 (1.06–1.25); <.001	71.5%	1.43 (1.30–1.58); <.001	88.6%
Mortality (adjusted)	32/36	1.04 (1.00–1.09); .033	16.1%	1.24 (1.16–1.33); <.001	76.2%
**Subgroup analyses based on adjusted mortality**					
**By gender**					
Both genders	20/23	1.04 (0.99–1.08); .096	11.1%	1.20 (1.11–1.29); <.001	79.0%
Men	8/8	1.13 (1.04–1.24); .007	0.0%	1.31 (1.10–1.58); .003	62.3%
Women	8/9	1.00 (0.85–1.18); .999	58.6%	1.48 (1.18–1.86); .001	80.4%
**By country**					
America	12/13	1.08 (1.03–1.14); .002	0.0%	1.19 (1.07–1.31); .001	78.2%
Europe	6/7	1.03 (0.93–1.14); .627	0.0%	1.01 (0.93–1.09); .823	0.0%
Asia	14/16	1.04 (0.96–1.12); .384	40.6%	1.41 (1.26–1.57); <.001	75.4%
**By total sleep time**					
Nighttime	19/23	1.05 (0.99–1.13); .113	17.8%	1.25 (1.13–1.38); <.001	73.7%
24 h	13/13	1.04 (0.99–1.10); .146	19.9%	1.25 (1.14–1.36); <.001	76.2%
**By ascertainment of sleep**					
Questionnaire	30/34	1.04 (1.00–1.09); .055	20.5%	1.26 (1.17–1.35); <.001	76.8%
Actigraphy	1/1	1.12 (0.89–1.42); .342	─*	0.83 (0.61–1.13); .233	─
Both	1/1	1.12 (0.75–1.68); .582	─	1.24 (0.73–2.10); .425	─
**By follow-up (years)**					
≥ 7.5	20/22	1.07 (1.02–1.12); .006	15.2%	1.24 (1.14–1.34); <.001	80.2%
< 7.5	13/14	0.99 (0.93–1.05); .736	0.0%	1.27 (1.12–1.45); <.001	68.3%
**By age**					
< 65	11/11	1.21 (1.02–1.23); .018	18.2%	1.38 (1.19–1.60); <.001	61.5%
≥ 65	21/25	1.03 (0.99–1.07); .193	4.2%	1.20 (1.11–1.30); <.001	78.2%
**Dose-analysis**					
≤ 5 h	15	1.06 (1.01–1.11); .014	12.3%	─	─
≤ 6 h	27	1.05 (1.01–1.10); .031	26.7%	─	─
≤ 7 h	32	1.04 (1.00–1.09); .033	16.1%	─	─
≥ 8 h	33	─	─	1.24 (1.16–1.33); <.001	77.9%
≥ 9 h	26	─	─	1.31 (1.21–1.41); <.001	71.9%
≥ 10 h	10	─	─	1.45 (1.24–1.70); <.001	82.6%

Abbreviations: HR, hazard ratio; 95% CI, 95% confidence interval. *Data are not available

### Publication Bias

**Fig. 2:**
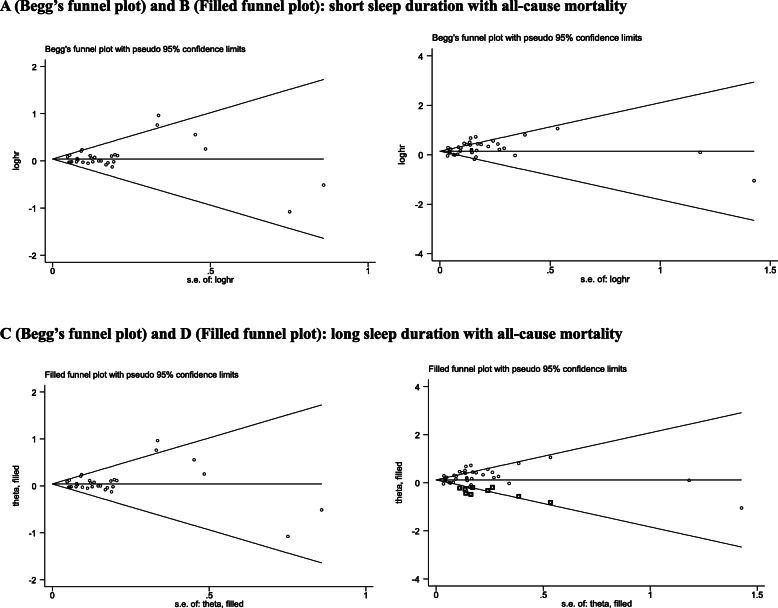
The Begg’s and filled funnel plots for the association of both short and long sleep duration with all-cause mortality

Figure 2 shows the Begg’s funnel plot to assess publication bias for the association of sleep duration with all-cause mortality, and only the plot of short sleep duration seemed symmetrical. As revealed by the Egger’s test, there was no evidence of publication bias for short sleep duration (*P* = .392), yet strong evidence of publication bias for long sleep duration (*P* = .020). Further filled funnel plots showed that there were 9 potentially missing studies due to publication bias to make the plot of long sleep duration symmetrical. After adjusting for these potentially missing studies, effect size estimates were still statistically significant for the association of long sleep duration with all-cause mortality (HR = 1.15; 95% CI: 1.07–1.23, *P* < .001).

### Subgroup analyses

A series of prespecified subgroup analyses were conducted to account for possible causes of between-study heterogeneity for both short and long sleep duration in the older people (Table 4).

By gender, the association of long sleep duration with all-cause mortality was statistically significant in both women (HR = 1.48; 95% CI: 1.18–1.86; *P* = .001) and men (HR = 1.31; 95% CI: 1.10–1.58; *P* = .003) (Two-sample Z test *P* = .205). By contrast, with regard to short sleep duration, statistical significance was observed in men (HR = 1.13; 95% CI: 1.04–1.24; *P* = .007), but not in women (HR = 1.00; 95% CI: 0.85–1.18; *P* = .999) (Two-sample Z test *P* = .099).

By geographic locations, the association of long sleep duration with all-cause mortality was stronger in Asia (HR = 1.41; 95% CI: 1.26–1.57; *P* < .001) than in Europe (HR = 1.01; 95% CI: 0.93–1.09; *P* = .823) (Two-sample Z test *P* < .001) and America (HR = 1.19; 95% CI: 1.07–1.31; *P* = .001) (Two-sample Z test *P* = .013). There was no observable difference for short sleep duration between Asia (HR = 1.04; 95% CI: 0.96–1.12; *P* = .384) and Europe (HR = 1.03; 95% CI: 0.93–1.14; *P* = .627).

By total sleep time, significance was only observed for the association of long sleep duration with all-cause mortality, and there was no material difference between the nighttime (HR = 1.25; 95% CI: 1.13–1.38; *P* < .001) and the 24 h sleep duration (HR = 1.25; 95% CI: 1.14–1.36; *P* < .001).

By ascertainment of sleep, for long sleep duration, the association was more evident for questionnaire survey (HR = 1.26; 95% CI: 1.17–1.35; *P* < .001) than for actigraph survey (HR = 0.83; 95% CI: 0.61–1.13; *P* = .233) (Two-sample Z test *P* = .004). Contrastingly, for short sleep duration, there was no detectable significance.

By the median value (7.5 years) of follow-up intervals, the association of long sleep duration with all-cause mortality was significant in both long (≥7.5 years) (HR = 1.24; 95% CI: 1.14–1.34; *P* < .001) and short (< 7.5 years) (HR = 1.27; 95% CI: 1.12–1.45; *P* < .001) follow-up. As for short sleep duration, the association was only significant in studies with long follow-up intervals (HR = 1.07; 95% CI: 1.02–1.12; *P* = .006).

By the median value (65 years) of baseline age, long sleep duration was significantly associated with all-cause mortality in both subgroups (≥65 years: HR = 1.20; 95% CI: 1.11–1.30; *P* < .001, and < 65 years: HR = 1.38; 95% CI: 1.19–1.60; *P* < .001), and for short sleep duration, only marginal significance was observed for studies with median age < 65 years (HR = 1.21; 95% CI: 1.02–1.23; *P* = .018).

### Dose-response analyses

**Fig. 3:**
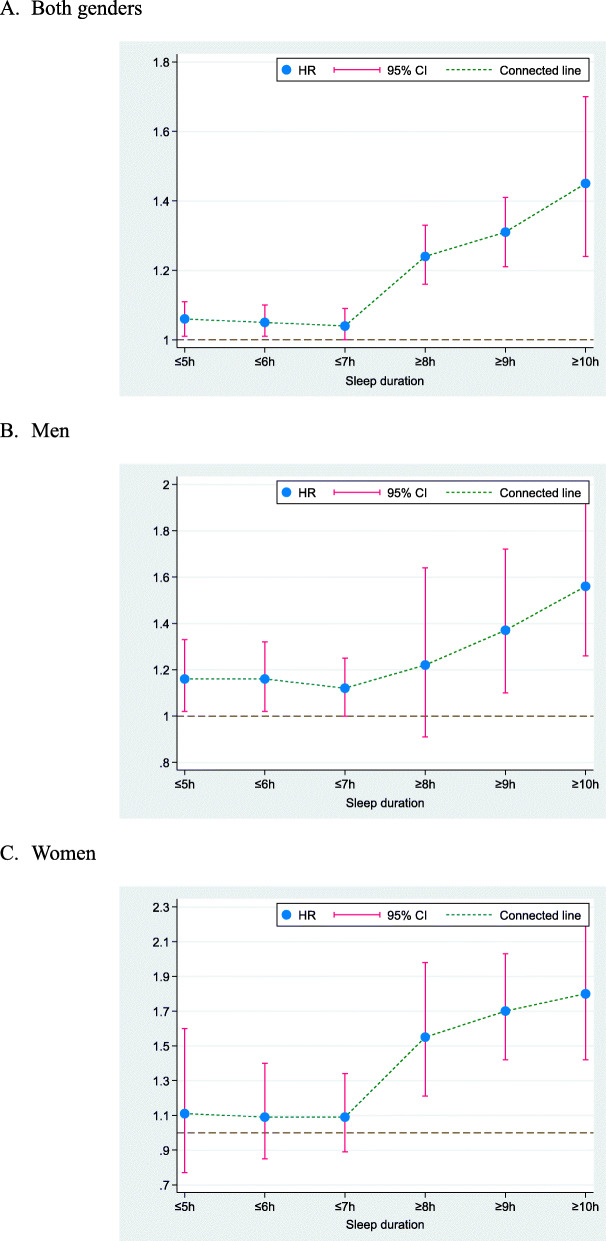
The trend plots of effect-size estimates with the increase of sleep duration in all older persons (A) and by genders (B and C). Abbreviations: HR, hazard ratio; 95% CI, 95% confidence interval

In the dose-response analysis on short sleep duration, all-cause mortality increased with the decrease of sleep time (≤5 h: HR = 1.06; 95% CI: 1.01–1.11; *P* = .014, ≤6 h: HR = 1.05; 95% CI: 1.01–1.10; *P* = .031, and ≤ 7 h: HR = 1.04; 95% CI: 1.00–1.09; *P* = .033) (Two-sample Z test *P* = .379 for ≤5 h vs. ≤6 h, and *P* = .379 for ≤6 h vs. ≤7 h) (Table 4). For long sleep duration, the trend was more evident (≥8 h: HR = 1.24; 95% CI: 1.16–1.33; *P* < .001, ≥9 h: HR = 1.31; 95% CI: 1.21–1.41; *P* < .001, and ≥ 10 h: HR = 1.45; 95% CI: 1.24–1.70; *P* < .001) (Two-sample Z test *P* = .147 for ≥8 h vs. ≥9 h, and *P* = .128 for ≥9 h vs. ≥10 h) (Table 4 and Fig. 3A).

In men, the risk associated with all-cause mortality was significant and increased with both shorter and longer sleep duration, and the increasing trend was more obvious for long sleep duration (Fig. 3B). In women, the risk associated with all-cause mortality was nonsignificant for short sleep duration, yet it was significantly increased with longer sleep duration in a graded manner, which was steeper than men (Fig. 3C).

**Fig. 4:**
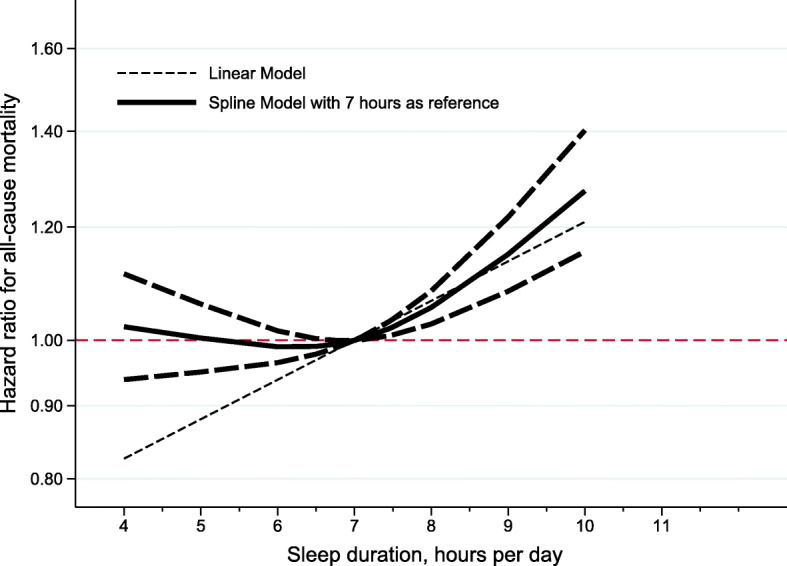
The dose-response relationship plot for the association of sleep duration with all-cause mortality. Lines with long dashes represent the pointwise 95% confidence intervals for the fitted nonlinear trend (solid line). Lines with short dashes represent the linear trend. The red horizontal line represents the reference line (hazard ratio: 1)

In both genders, dose-response regression analyses, using log (effect-size estimates) as dependent variable and categorized sleep duration as independent variable, revealed that trend estimation was more obvious for long sleep duration (regression coefficient: 0.13; *P* < .001) than for short sleep duration (regression coefficient: 0.02; *P* = .046) (Fig. 4). In men, the regression coefficient for tread estimation was 0.05 (*P* = .022) and 0.15 (*P* < .001) for short and long sleep duration, respectively, and the regression coefficient was separately 0.04 (*P* = .449) and 0.20 (*P* < .001) in women.

## Discussion

To the best of our knowledge, this is thus far the most comprehensive meta-analysis that has explored the dose-response relationship between sleep duration and all-cause mortality in the older people. It is worth noting that long sleep duration was associated with a significantly increased risk of all-cause mortality, especially in women, and the mortality risk associated with short sleep duration was only significant in men. Moreover, besides gender, geographic region, sleep survey method, baseline age and follow-up interval were identified as possible causes of between-study heterogeneity. Our findings highlight the importance and the necessity of closely monitoring the sleep status of elders who have long sleep duration, as well as elderly men of sleep deficiency, to control and prevent all-cause mortality.

In the previous meta-analysis of 27 cohort studies by da Silva and colleagues, both long and short sleep duration were found to be associated with a significantly increased risk of all-cause mortality risk in the older people [18]. Differing from the meta-analysis by da Silva and colleagues [18], we restricted analysis only to prospective cohort studies that reported HRs and 95% CIs to quantify the association between sleep duration and all-cause mortality in elders. After synthesizing the adjusted effect-size estimates from 28 articles including 95,259 older persons, albeit the consistent marginal significance for short sleep duration in overall analyses, extending the findings by da Silva and colleagues [18], we in subsidiary analysis observed a remarkably significant mortality risk associated with short sleep duration in men only. Similarly, da Silva and colleagues [18] and we unanimously supported the significant contribution of long sleep duration to all-cause mortality. The reasons behind above inconsistent observations are manifold. First, the most likely reason is the unaccounted confounding, as our analysis based on unadjusted effect-size estimates indicated that short sleep duration was a significant predictor for all-cause mortality, yet no significance was detected after adjustment.

Another possible reason is the synthesis of different types of effect-size estimates. To minimize this statistical noise, we restricted analysis to only HRs that were calculated after adjusting for confounding factors, despite the varying panels of adjusted factors across each involved study in this meta-analysis. The third reason is the significant heterogeneity across individual studies. To fully account for this, we conducted both subgroup and meta-regression analyses, and found that gender, geographic region, sleep survey method, baseline age and follow-up interval were possible causes of between-study heterogeneity. We agree that future large-scale, well-designed cohort studies were warrant to derive a relatively reliable estimate.

Although the mechanisms for the association between long sleep duration and all-cause mortality are not completely understood, the current possible explanation is that sleep affects the human body through inflammatory processes. When sleep duration is too long, concentrations of inflammatory markers, such as interleukin-6 and C-reactive protein can increase [48, 49]. In addition, it is reported that unstable sleep duration was associated with some common diseases, such as hypertension [50, 51], diabetes [52], and coronary heart disease [53, 54]. It is hence reasonable to speculate that long-term irregular sleep duration is likely to destroy the body’s immune system balance through chronic inflammatory processes, and further increase all-cause mortality risk. There is also evidence showing that sleep has a crucial impact on autonomic nervous system, system dynamics, cardiac function, endothelial function and coagulation [55]. Nevertheless, over sleep duration can accelerate the occurrence or progression of chronic diseases, and further precipitate all-cause mortality.

It is worth noting that we identified strong evidence of between-study heterogeneity for the association of long sleep duration with all-cause mortality, irrespective of adjustment. By contrast, for short sleep duration, heterogeneity was dwindled from strong in the unadjusted model to low in the adjusted model. It is hence reasonable to expect that besides methodological heterogeneity (such as study design), clinical heterogeneity like different baseline characteristics (such as age, sex ratio, dietary habits) of study populations in this meta-analysis may explain the discrepancy. In particular, insufficient adjustment for residual confounding by incompletely measured or unmeasured clinical covariates might exist in our results. As such, translating our findings into clinical practice should be done with caution.

Finally, some limitations should be acknowledged for this present meta-analysis. First, only sleep duration was considered in this study, and other sleep-related indexes, such as sleep quality, are of added interest for explorations in case of sufficient eligible studies. Second, although adjusted effect-size estimates were synthesized in this meta-analysis, some important confounding factors are still not taken into account by all involved studies, such as physical activity and other lifestyle factors. For example, in a long-term follow up of older adults in the UK, physical activity and prefrailty was observed to be significant modifiers for the prediction of long sleep duration for all-cause mortality [40]. Third, although there was a high probability of publication bias for long sleep duration as reflected by Begg’s funnel plot and Egger’s test, we adopted the trim-and-fill method to impute theoretically missing studies and recalculated our pooled effect-sized estimate, which was still statistically significant. Fourth, although a large panel of subgroup and meta-regression analyses were undertaken to account for possible causes of heterogeneity, significant heterogeneity still persisted in some subgroups, limiting the interpretation of pooled effect-size estimates. Last but not the least, the majority of studies involved in this meta-analysis recorded sleep duration based on nighttime, and data on naps are sparse.

## Conclusions

Taken together, our findings indicate a significantly increased risk of all-cause mortality associated with long sleep duration, especially in women, as well as with short sleep duration in men only. We agree that the findings of this meta-analysis pose a challenging task for searchers, clinicians, and policy makers to attach importance to monitor the sleep status of elders, especially with long sleep duration. Further investigations on the molecular mechanisms linking sleep duration and all-cause mortality are also warranted.

## Supplementary information

**Additional file 1.**
